# Autophagy and Cancer

**DOI:** 10.3390/cells1030520

**Published:** 2012-08-13

**Authors:** Francesca Aredia, Luis Miguel Guamán Ortiz, Vincenzo Giansanti, A. Ivana Scovassi

**Affiliations:** 1 IGM-CNR, Via Abbiategrasso 207, Pavia 27100, Italy; Email: francesca.aredia@gmail.com (F.A.); vincenzogiansanti84@libero.it (V.G.); 2 UTPL, Loja 1101608, Ecuador; Email: lmguaman@utpl.edu.ec

**Keywords:** apoptosis, autophagy, cancer, cell death

## Abstract

Autophagy is a housekeeping survival mechanism with a protective function against stress conditions. However, when stress severity or duration increases, it may promote cell death. Paradoxically, autophagy favors cancer development, since cancer cells could enhance their proliferation potential (thus becoming able to resist anticancer therapy) thanks to the energetic supply provided by organelle degradation typically driven by autophagy following a stepwise pathway. The main actors of the autophagic machinery as well as the features shared with apoptosis will be described. Special attention will be paid to the effects of autophagy manipulation.

## 1. Introduction

Cancer is one of the multifactorial and multistep complex disorders that accounts for a major cause of death all over the world, accounting 7.6 million deaths (around 13% of all deaths) in 2008 [1]; it is characterized by uncontrolled proliferation of abnormal cells that ends with the formation of a tumor. During tumor development, cancer cells can acquire many features such as sustained proliferative signaling (active oncogenes), evasion of growth suppressor functions, invasion of healthy tissues due to metastatic potential, replicative immortality, angiogenesis stimulation and resistance to cell death induced by chemotherapeutic agents [2,3]. For decades, the scientific community has been working to understand not only the molecular mechanisms at the basis of the uncontrolled proliferation of cancer cells, but also how these cells become insensitive to internal/external stimuli promoting cell death. Drug resistance of cancer cells is often correlated to an impaired activation of Programmed Cell Death (PCD), mainly occurring through the apoptotic pathway(s); accordingly, it has been assumed for a long time that the re-activation of apoptosis could be sufficient to promote the eradication of cancer cells [4,5]. Classical apoptosis implies the activation of caspases, which are in charge for extensive protein degradation [6]; this event could be also triggered by the release of proteolytic enzymes from lysosomes (lysosomal-mediated cell death) [7]. Moreover, necroptosis, “an ordered cellular explosion”, represents a cell death mechanism with morphological features resembling necrosis [8]. The scenario is even more complicated, given that a housekeeping process, *i.e.*, autophagy, which regulates physiological functions, could also promote cancer cell survival, as illustrated below [9,10,11].

## 2. Main Features of Autophagy

The term Autophagy comes from the Greek words *αύτος* (*autos*) and *φαγέω* (*fageo*), which means “*self-eating*”, a catabolic self-degradation process for maintaining normal cell homeostasis to ensure the regular turnover of cellular components [12]. Four types of autophagy have been described in mammals: Micro-autophagy (MicroA), Chaperone-mediated autophagy (CMA), Macro-autophagy (MacroA) and Alternative Macro-Autophagy (AMA) [13]. Autophagy can selectively target organelles such as mitochondria (Mitophagy), ribosomes (Ribophagy), peroxisomes (Pexophagy), and endoplasmic reticulum (ER; Reticulophagy), thus contributing to their turnover (reviewed in [14]).

Autophagy is tightly regulated by a limited number of highly conserved genes called ATG (AuTophaGy related genes) that were first identified in *Saccharomyces* *cerevisiae* [15,16,17]. This finding facilitated the discovery of mammalian orthologues and the further definition of the autophagic machinery in other organisms [18]. Autophagy is considered as a survival mechanism, having a protective function in many cellular stress conditions [19,20,21], through the ability to counteract nutrient deprivation by recycling energy originated from macromolecule degradation. In case of prolonged starvation conditions, cells “eat” part of their own cytoplasmic components to compensate the lack of metabolites needed to synthesize essential molecules [22]. However, when stress severity or duration is extended, autophagy may participate in cell death such as type II PCD [23].

## 3. Execution of Autophagy

**Figure 1:**
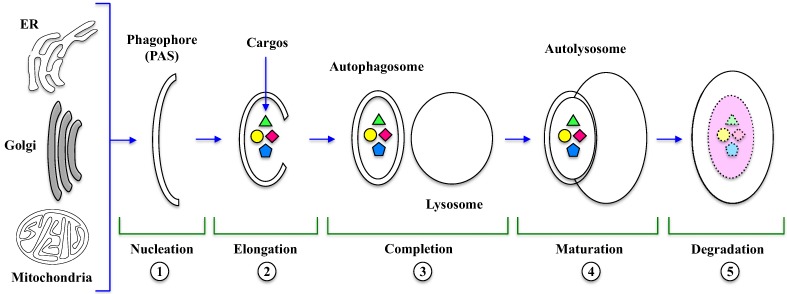
*Autophagosome* *and* *autolysosome* *formation*. Several morphological changes occur during autophagy, which is stepwise regulated. In the *Nucleation* (1) and *Elongation* (2) steps, phagophore originates from membranes of organelles (ER, Golgi, mitochondria) and then encloses the cytosolic cargos, including long-lived, misfolded proteins and damaged organelles, leading to the formation of the autophagosome *Completion* (3). The *Maturation* (4) step consists in the fusion of autophagosome with lysosome to form the autolysosome. Finally, during *degradation* (5), lysosomal hydrolases digest autolysosomal content and release products in the cytosol. PAS: Phagophore Assembly Site; ER: endoplasmic reticulum.

The key event in autophagy is the formation of autophagosome and autolysosome, a process that requires several sequential steps illustrated in Figure 1.

**Figure 2:**
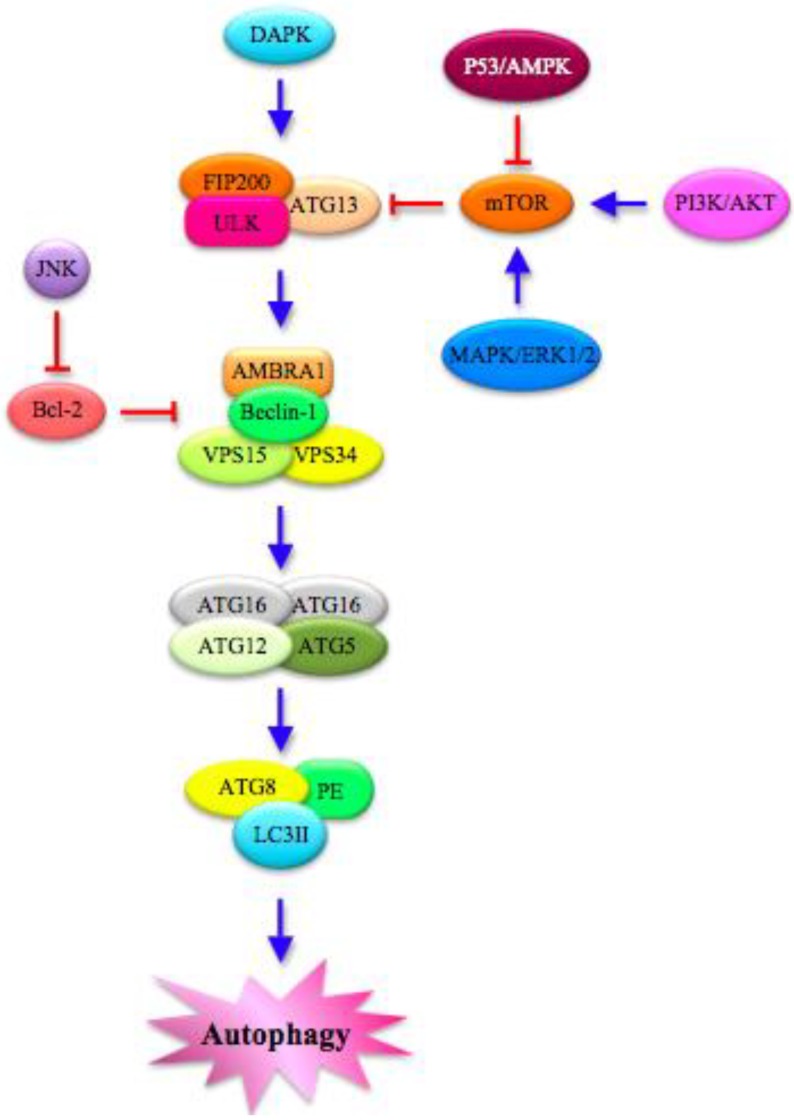
*Autophagy* *pathway:* *Molecular* *features*. Several proteins in the cytoplasm interact to regulate autophagy. The different steps of the process are modulated by MAPKs (Mitogen-Activated Protein Kinases), such as ERK (Extracellular Signal-Related Kinase) 1/2 and JNK (c-Jun N-terminal Kinase), which mainly act on the key negative regulator of autophagy mTOR (mammalian Target OF Rapamycin). mTOR is also controlled directly by PI3K/AKT (PhosphatidylInositol 3-Kinase/Protein Kinase B) and p53/AMPK (AMP-Activated Protein Kinase) signaling pathways. Moreover, DAPK (Death-Associated Protein Kinase) is implicated in the autophagic cascade, which starts with the formation of ULK (Unc-51-Like Kinase) complex, which is composed of FAK (Focal Adhesion Kinase)-family Interacting Protein of 200 kDa (FIP200), ULK and ATG13. In turn, ULK complex phosphorylates AMBRA1 (Activating Molecule in Beclin-1-Regulated Autophagy), leading to the activation of a complex that includes P-AMBRA1, Beclin-1, VPS (Vacuolar Protein Sorting) 15 and 34. The final steps are characterized by the assembly of ATG complexes made by ATG factors and autophagosome proteins. PE: Phosphatidyl Ethanolamine; LC3II: lipidated form of Microtubule-Associated Protein Light Chain 3.

The first event is *nucleation* (1), where a double-membrane structure called Phagophore is formed, which derives mainly from endoplasmic reticulum, Golgi, endosomes and even mitochondria and plasma membrane [24]. In this initial step, an ubiquitin-like system regulates the formation of ATG5-ATG12 heterodimer (Figure 2), which, in the presence of an ATG16 homodimer, forms a protein complex and associates to the Phagophore Assembly Site (PAS) [24].

In the next step, PAS expands by direct flow from a source (e.g., ER) and then seals to enclose the cytosolic cargos like long-lived, misfolded proteins and damaged organelles [12]. Ubiquitin-like (Ubl) conjugation systems are involved in vesicle *elongation* (2) and authophagosomal membrane *completion* (3). The mammalian orthologue of yeast ATG8, called LC3-I (Microtubule-Associated Protein Light Chain 3) is conjugated to the lipid PhosphatidylEthanolamine (PE), whereas ATG12 is conjugated to ATG5 [25]. At this stage, LC3-I is first cleaved, then lipidated to form LC3-II, which is incorporated into the nascent structure; for this reason, the presence of LC3-II is the most specific marker for autophagosome formation and, more in general, for autophagy occurrence. Then, the autophagosome fuses with the lysosome to form the autolysosome in a process called *maturation* (4) [24,25], controlled by cytoskeleton and lysosome membrane proteins [26].

The autophagosome conversion into autolysosomes can be blocked by edazol, a drug that specifically target microtubules [27]. The final step is the *degradation* (5) of autolysosomal content by lysosomal hydrolases that metabolize lipids, sugars, proteins and nucleotides; the degradation products are released in the cytoplasm and can be reutilized or become an energy source [24,25,28].

The entire process is tightly regulated by a cascade of kinases (Figure 2). Mitogen-Activated Protein Kinases (MAPKs), including ERK (Extracellular Signal-Related Kinase) 1/2, p38 and JNK (c-Jun N-terminal Kinase), play a fundamental role in governing the key negative regulator of autophagy mTOR (mammalian Target Of Rapamycin), a conserved Ser/Thr kinase [29,30]. DAPK (Death-Associated Protein Kinase), PI3K/AKT (Phosphatidylinositol 3-kinase/Protein Kinase B) and p53/AMPK (AMP-Activated Protein Kinase) signaling pathway also mediate the induction of autophagy through the modulation of mTOR [9,10,25,29,31,32].

Under stress conditions, mTOR is inactivated, thus allowing autophagy to start through the formation of the ULK complex (Figure 2), composed of FAK (Focal Adhesion Kinase) -family Interacting Protein of 200 kDa (FIP200), Unc-51-Like Kinase (ULK) and ATG13 [33]. In the proximity of the phagophore, a multimeric PI3K (PhosphatidylInositol 3-kinase) complex is also formed, which is controlled positively by UV radiation Resistance-Associated Gene (UVRAG) and negatively by Rubicon [34]. This complex includes Beclin-1 (released at the ER level), Vacuolar Protein Sorting (VPS) 15, VPS34 and Activating Molecule in Beclin-1-Regulated Autophagy (AMBRA1) [34]. Beclin-1, a member of the Bcl-2 family and the mammalian homolog of the yeast ATG6 gene, is positively regulated by AMBRA1, which is phosphorylated and released from the dynein motor complex during autophagy initiation [35]. In addition, the dissociation of the Beclin-1/Bcl-2 complex can be promoted by p53 as an apoptotic response inhibiting the mTOR signal [36].

## 4. The Paradoxical Role of Autophagy in Cancer

A multitude of internal and external stimuli can persuade a healthy cell to become malignant. Once this process is activated, a series of biochemical events drive an uncontrolled proliferation status. The new progeny of transformed cells first has to evade cell death in order to sustain chronic proliferation and consolidate the tumor microenvironment. Autophagy deregulation is prevalent in many cancers and involves several autophagic genes or proteins (reviewed in [36,37]). For instance, in 40%–75% of human breast, ovarian, and prostate cancers the Beclin-1 gene is monoallelically deleted [38]. In gastric and colorectal cancers, UVRAG and others ATG genes, showed frameshift mutations [39,40,41]. Conversely, the autophagic marker LC3 is highly expressed in more than 50% of human gastric cancers [42]. Table 1 reports representative examples of the altered status of autophagic factors.

**Table 1:** Deregulation of some autophagic factors in human cancers.

Gene	Mutation	Cancer	Reference
*Beclin-1*	allele deletion decreased expression	breast, ovarian, prostatic liver	[38,43,44]
*LC3*	increased expression downregulation	gastric, esophageal melanoma	[42,45]
*UVRAG*	frameshift mutations	gastric	[39]
*ATG8*	increased expression	colorectal	[40]
*ATG2B*, *ATG5*, **ATG9B	frameshift mutations	colorectal, gastric	[41]

Even in cancer cells, as it occurs during normal homeostasis control, autophagy senses stress signals and promotes the lysosomal degradation of organelles and proteins. The impact of autophagy in the yet complex network governing cancer progression could occur at different levels: (i) counteracting genome instability, thus impairing malignant transformation; (ii) protecting cancer cells from unfavorable conditions, thus promoting tumorigenesis [9,10].

(i) Autophagy can act as a tumor suppressor by removing damaged organelles and growth factors, and by facing chromosomal instability. In this respect, the autophagy factors Beclin-1 and Atg5 are considered as ‘guardians’ of the cellular genome. Mathew *et* *al.* [46] demonstrated that immortalized epithelial cells with loss of Beclin-1 or Atg5 have increased DNA damage, gene amplification and aneuploidy, in parallel with enhanced tumorigenicity. In addition, they found that defective autophagy (*Beclin-1*^+/−^ and A*tg5^−/−^*) in immortalized Baby Mouse Kidney (iBMK) cells caused the accumulation of p62 protein aggregates, damaged mitochondria and misfolded proteins, driving the production of Reactive Oxygen Species (ROS) [47]. The active role of UVRAG in maintaining genomic stability has been demonstrated in *UVRAG* depleted cells, which were affected in centrosome stability, chromosome segregation and spindle formation [48]. This body of evidence suggests that appropriate protein quality control by autophagy contributes to contrast tumorigenesis.

(ii) Paradoxically, the cytoprotective role generally played by autophagy can be harmful in cancer cells, as it can help them resist anticancer therapy [49,50]. In fact, autophagy not only provides energy for cell division, but also has a role in eliminating damage caused by tumor microenvironment and anticancer therapies (reviewed in [51]). Indeed, several reports showed that hypoxia, a common condition in solid tumors, activates autophagy in cancer cells [52,53]; analogously, extracellular pH changes occurring during cancer development modulate autophagy [54]. Under these non-physiological conditions, autophagy responds by degrading damaged organelles, DNA and proteins and providing cancer cells with new energy, useful to sustain their proliferation. Furthermore, autophagy has been reported to increase cell survival during anoikis, which is the first step in the process of cancer cell metastatization, thus giving an advantage to migrating dangerous cells [55]. The relevance of autophagy for cell invasion is supported by the observation that the knockdown of *Atg12*, and the consequent inhibition of the autophagic machinery, decreases the invasiveness of glioma cells [56]. For the above reasons, autophagy could exert a dangerous function in cancer.

A further level of complexity is added by the role of p53, which, in addition to the direct control of DNA repair, also tunes the autophagic burst acting as a “rheostat” that continuously adjusts the rate of autophagy with the final aim of serve as an anticancer mechanism [57]. To do this job, p53 downregulates post-transcriptionally the autophagic protein LC3, thus keeping autophagic flux at sustainable level and avoiding excessive autophagy, potentially favorable to abnormal cancer cell proliferation [58]. However, depending on genetic and epigenetic features (e.g., p53 mutations or inactivation), the oncosuppressor effect of p53 could be abolished, rendering autophagy free from oncosuppressor control.

## 5. Autophagy Crosses Apoptosis

The adverse phenomenon of drug resistance often occurring in cancer cells has been correlated to an alteration of PCD machinery, mainly of apoptosis [4,5]. However, it has recently been shown that a housekeeping process, *i.e.*, autophagy, could modulate cancer cell survival [11]. Apoptosis and autophagy are not independent processes, given that a complex crosstalk between them has been depicted, leading to the notion that they can be triggered by common upstream signals and share molecular switches [9,10,59].

Proteins that are central components of apoptosis or autophagy machinery can regulate both processes directly. Beclin-1 and Bcl-2 family members represent the best example: Bcl-2 and Bcl-X_L_ inhibit Beclin-1 by binding to it through the Beclin-1 BH3 domain [60,61], which is atypical, lacking a hydrophobic aminoacid at position 119 (which corresponds to the polar Thr) [60,62]. This feature lowers Beclin-1 affinity for Bcl-2 compared to other BH3-containing proteins. When autophagy is essential for cell survival, the association between Bcl-2 and Beclin-1 decreases, thanks to the action of JNK-1 on Bcl-2, thus allowing Bcl-2 dissociation from Beclin-1 and autophagy promotion. Phosphorylated Bcl-2 is now free to bind the pro-apoptotic protein Bax, thus inhibiting apoptosis, which, in such context, would be promoted by loss of nutrients [63,64]. However, under extreme conditions, JNK1 hyper-phosphorylates Bcl-2, which detaches from Bax, thus facilitating apoptosis and consequently a safe cell death [63,64]. Given that the affinity of phosphorylated Bcl-2 toward pro-apoptotic proteins is higher than toward Beclin-1, a model has been hypothesized to explain Bcl-2 binding properties [63,64]. Analogously, DAPK phosphorylates Beclin-1 on Thr119, thus promoting its dissociation from Bcl-2, and autophagy activation [65]; DAPK is also involved in apoptotic bleb formation thanks to its interplay with cytoskeletal factors [66].

ERK is a kinase implicated in both apoptosis and autophagy [67,68]. ERK pathway plays a critical role in promoting apoptosis in response to several stress stimuli, both intrinsic and extrinsic. In addition, when ERK is phosphorylated, it acts as the main switch between apoptosis and autophagy, e.g., phosphorylating the α subunit of the eukaryotic Initiation Factor 2 (eIF2) to attenuate protein synthesis [69,70]. This modification determines whether the cell fate is switched to autophagy, by means of ATG5-12 complex-activated LC3, or to apoptosis through caspase activation.

Another protein strictly involved in the crosstalk is ATG5, which, other than promoting autophagy, has a role in enhancing apoptotic stimuli. In fact tumor cells overexpressing *Atg5* were reported to be more sensitive to chemotherapy, while in case of gene silencing, cancer cells were partially resistant to anti-cancer drugs [71]. This occurs because ATG5, during apoptosis, is cleaved by calpains and subsequently translocated to mitochondria, where it interacts with Bcl-X_L_ and controls cytochrome c release and caspase activation [72]. ATG3, controlled by FLICE-Inhibitory Protein (FLIP), is involved also in apoptosis, where it regulates negatively the extrinsic pathway by recognizing and binding FADD (Fas-Associated protein with Death Domain) through specific Death Effector Domains (DED). An additional aspect that supports the interplay between apoptosis and autophagy is the involvement of apoptotic caspases in the degradation, and consequent inactivation, of autophagic proteins such as Beclin-1, thus inhibiting autophagy and consequently enhancing apoptosis progression (reviewed in [9,10]). In addition, ATG12 could play an active role in both processes [73].

Several reports highlighted the role of p62 (also called sequestosome), a protein that targets other proteins for proteasome degradation or autophagic digestion, at the crossroads of autophagy, apoptosis and cancer [74]; in particular, it has been shown that LC3-II binds p62 to regulate protein packaging and delivering to the autophagosome [75,76]. In addition, p62 accumulation was described in autophagy-defective cells, which suffer from proteasome inactivation and altered NF-kB regulation, and undergo tumorigenesis [47]. This aspect is in agreement with the observation describing how p62 is involved in apoptosis: in the extrinsic pathway, the initiator caspase-8 requires p62 for its efficient polyubiquitination, aggregation and full activation [77].

Notably, it has recently been reported that autophagosome is required to activate caspase-8, which is able to interact with some autophagic factors such as p62 and ATG5; in particular caspase-8/FADD complex associates with ATG5 on ATG16- and LC3-positive structures, suggesting the role of the autophagosomal membrane as a platform for the formation of a dual-armed DISC (Death-Inducing Signaling Complex) that facilitates the activation of caspase-8 and initiation of apoptosis [78]. Conversely, it has been found that caspases cleave and inactivate Beclin-1, thus inhibiting autophagy and enhancing apoptosis, given that the proteolytic Beclin-1 C-terminal fragment induces the release of apoptotic factors from mitochondria [79,80,81]. These observations strongly support the dependence of apoptosis from autophagy and *vice versa* [78] and further stimulates the discussion about the real distinct identity of these processes [82].

## 6. Autophagy Manipulation

The intricate relation between apoptosis and autophagy leads to opposite situations: suppression of apoptosis could induce autophagy, while autophagy inhibition causes apoptosis [9,10,60,83].

**Figure 3:**
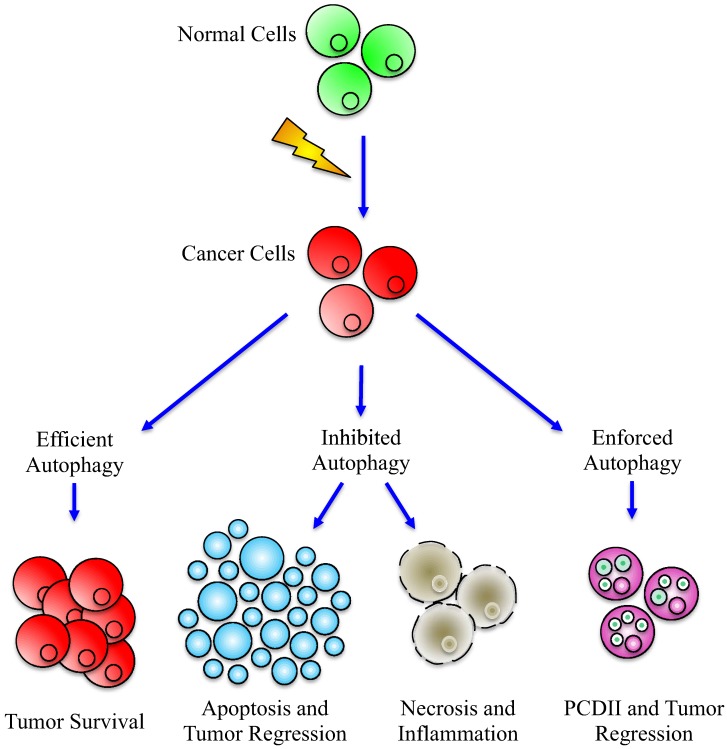
*Effects* *of* *efficient* *or* *deregulated* *Autophagy* *in* *cancer* *development*. Alteration of autophagy may have different effects on cancer cells: when efficient, the autophagic machinery could help cancer cells to survive and proliferate (left part), while, when inhibited, autophagy cannot anymore sustain cancer progression, leading to the activation of apoptosis or to necrosis (central part) and, by consequence, tumor regression. Paradoxically, the same end point can be reached after enforced activation of autophagy (right part), which can act as type II PCD (Programmed Cell Death).

The complex impact of autophagy on cancer cell metabolism is schematized in Figure 3. A possible way to take advantage of autophagy manipulation is the idea of battery-operated tumor growth, according to which inhibiting or forcing autophagic machinery would be useful in drug cancer treatment [84].

The possibility to manipulate autophagy for fighting cancer is extremely intriguing: many groups attempted to sensitize cancer cells to treatments through the use of inhibitors/activators of the autophagic machinery [85]. In this respect, on the one hand, inhibitors of autophagy applied in combination with anticancer agents could improve the efficacy of classical drugs; on the other hand, activators of autophagy could enforce the cell death potential of autophagy itself.

For example, it has been shown that after anti-angiogenesis therapies, cancer cells respond to hypoxia by activating autophagy and, in this way, they can survive rendering the therapy no more effective [86]. This observation suggests that the inhibition of autophagy may cooperate with anti-angiogenic factors to avoid drug resistance. Conversely, the activation of autophagy could represent a powerful strategy to kill cancer cells, as reported for some Triple-Negative Breast Cancer (TNBC) -derived cell lines, where the treatment with an mTOR inhibitor kills cancer cell, providing the evidence that the release of autophagy inhibition could be useful to counteract cancer growth [87].

These reports are representative (and contradictory) examples of the rationale basis for manipulating autophagy in order to interfere with cancer cell metabolism. An exhaustive list of the methods for developing autophagy-based therapies, together with “Pros and Cons” of these strategies, has been recently drawn [36,49,85 and references therein]. On the whole, the knowledge of the molecular bases of autophagy has encouraged many attempts to modulate autophagy in order to identify new tools for elaborating an efficient action plan against cancer. In fact, the existence of more than 20 ongoing clinical trials based on inhibition/stimulation of autophagy [88] supports the growing interest toward the impact of this process and the possible applications in clinics.

As a further matter of debate, although the development of autophagy-based anticancer strategies is promising, this approach has to be carefully examined with respect to undesirable effects on non-cancer cells [36]. On the one hand, strategies able to manipulating autophagy could be a novel weapon to treat cancer; on the other hand, they could have a noxious impact on normal cells, such as neurons. For example, it has been shown that genetic inhibition of autophagy allows the occurrence of neurodegenerative hallmarks possibly culminating in aging [89,90]. In fact, the role of autophagy in neurodegenerative disorders has not been fully elucidated, being either beneficial or detrimental, depending on the disease features [91,92]. Notably, attempts to pharmacologically modulate autophagy through the use of the mTOR inhibitor rapamycin (and analogs), allowed neuroprotection in several experimental models of neurodegenerative diseases, due to the contribute to the clearance of intracellular protein aggregates [93,94,95]. In this respect, autophagic dysfunction has been described in neurodegenerative disorders, thus opening new perspectives for clinical treatments [96].

## 7. Conclusions

The aim of this review is to delineate the impact of the so complex and amazing autophagic process on cancer cell survival. In this respect, the investigation of the role played by autophagy in the complex network of cell death(s) as well as the in-depth examination of its intricate connection with apoptosis, could help in understanding when and how autophagy switches from a survival to a death function [92]. Taking into account the two faces of Janus of autophagy [91], the attempt to define the autophagic function in a univocal manner is a hard duty. This housekeeping process, due to its enrollment in several basic functions, assumes more than one connotation, and for this reason it can be defined only within the frame of the specific biological context [51,97,98]. The data collected in this field could be crucial in defining the cascade of events transforming a normal cell in a tumoral one and eventually confer metastatic potential to it. In conclusion, the “take home message” from the above considerations is that the frontier between the opposite functions of autophagy is not sharp but multi-faceted and depends on the subtle regulation of survival/death signals, including those governing cancer development, drug resistance and neuronal deficits.

## Supplementary Files

Supplementary File 1 ZIP-Document (ZIP, 7225 KB)
