# Supplementary figures and images for: Autophagy and Cancer
Source: Cells. 2012 Aug 13;1(3):520–34. doi: 10.3390/cells1030520 (PMC3901115; doi:10.3390/cells1030520)

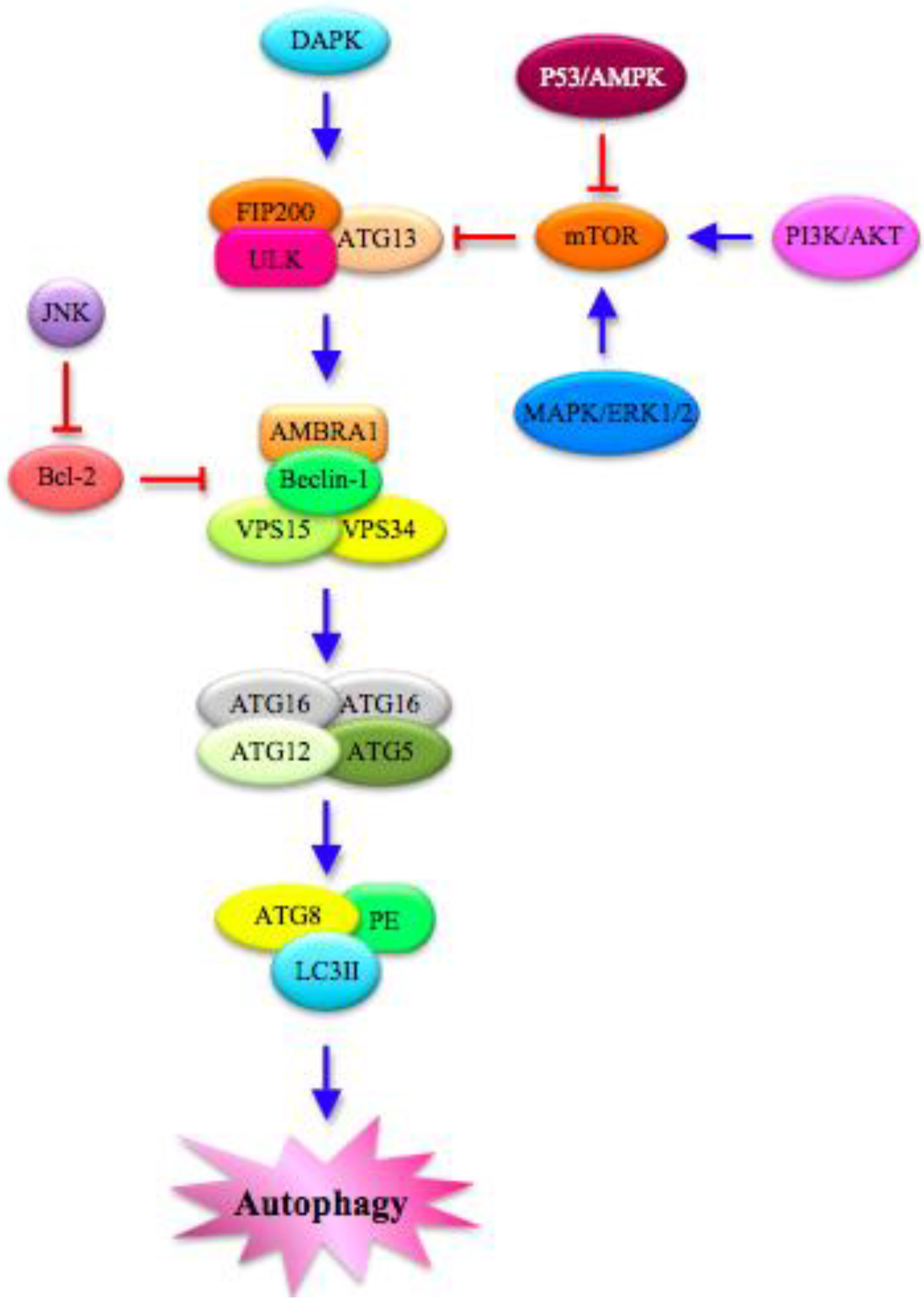

Supplement: Supplementary File 1 — ZIP-Document (ZIP, 7225 KB) [file cells-01-00520-s001.zip › cells-01-00520-g002.tif]

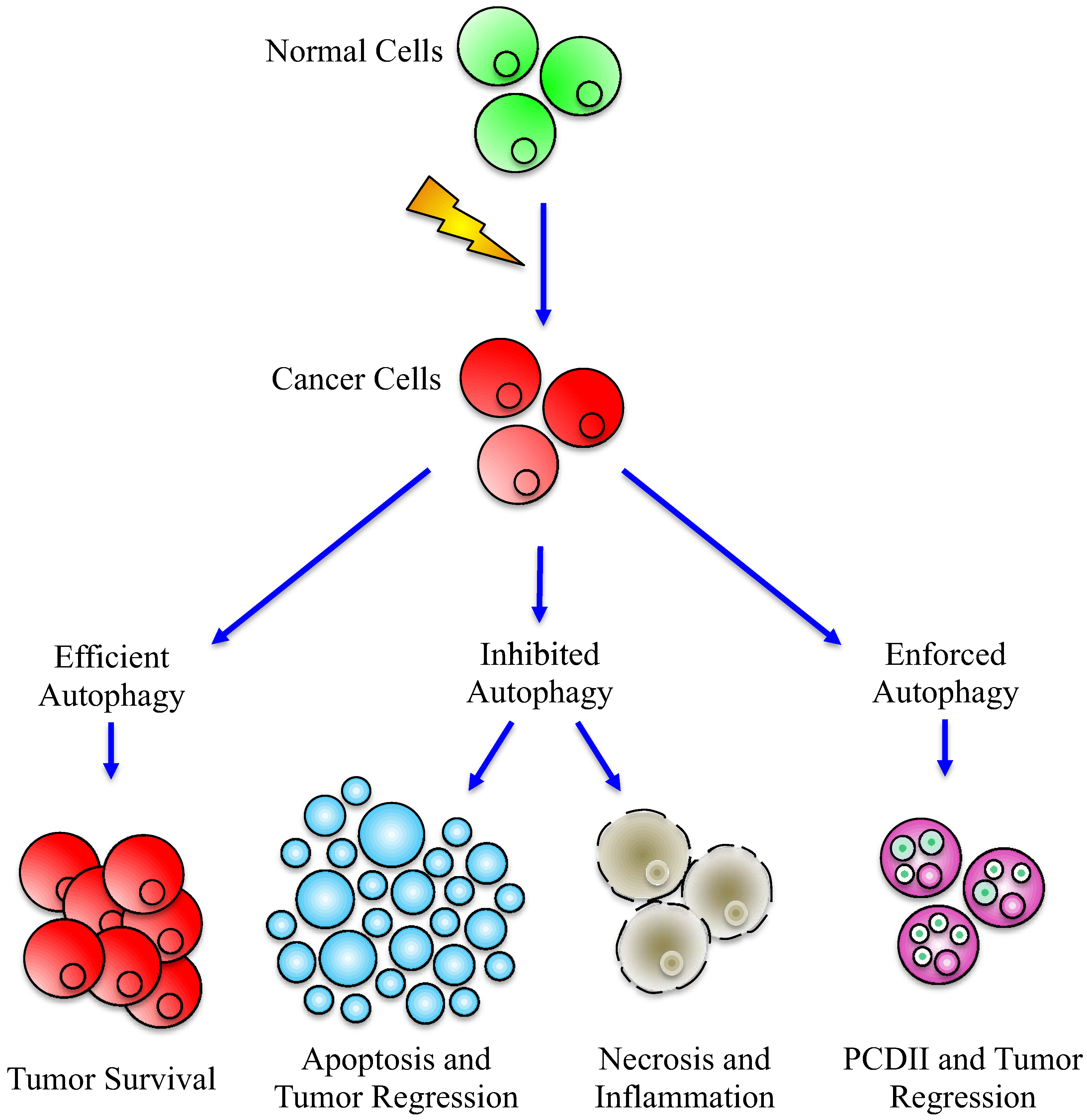

Supplement: Supplementary File 1 — ZIP-Document (ZIP, 7225 KB) [file cells-01-00520-s001.zip › cells-01-00520-g003.tif]

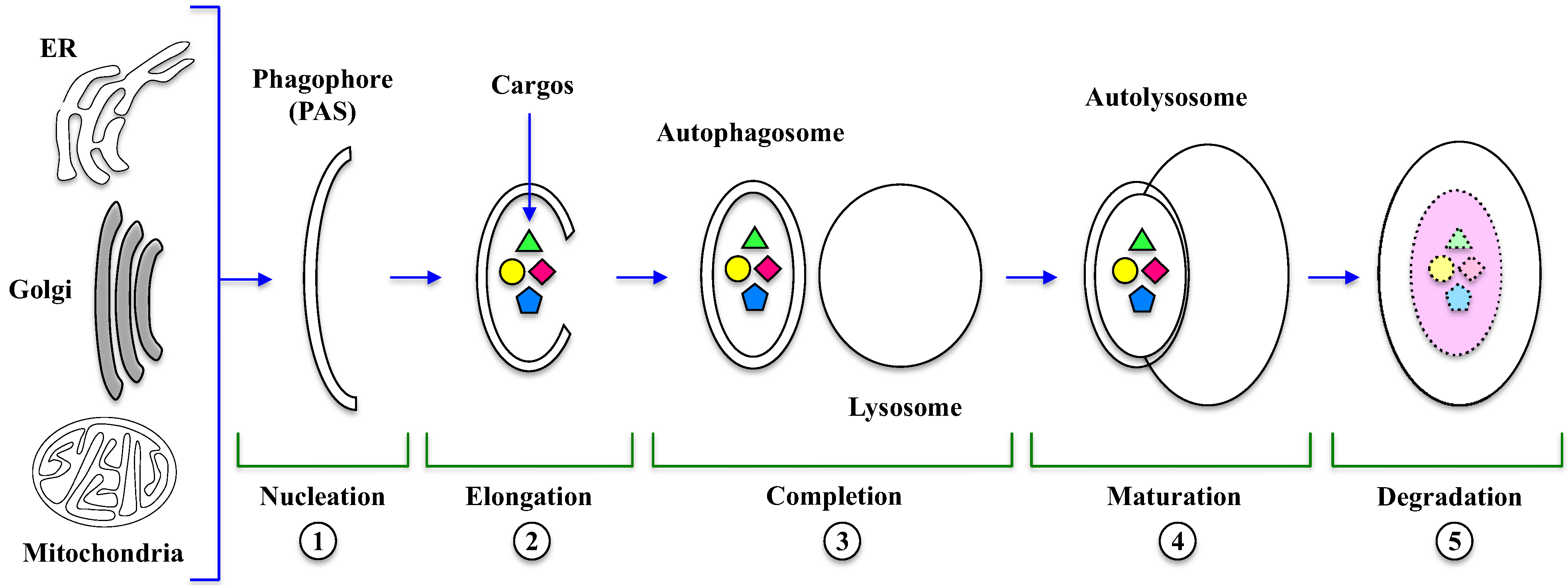

Supplement: Supplementary File 1 — ZIP-Document (ZIP, 7225 KB) [file cells-01-00520-s001.zip › cells-01-00520-g001.tif]
